# Hirntodkriterium und Organspende: aktuelle neurowissenschaftliche Perspektive

**DOI:** 10.1007/s00103-020-03245-1

**Published:** 2020-11-12

**Authors:** Uwe Walter

**Affiliations:** 1 Klinik und Poliklinik für Neurologie, Universitätsmedizin Rostock, Gehlsheimer Str. 20, 18147 Rostock, Deutschland; 2 Centre for Transdisciplinary Neurosciences Rostock (CTNR), Universität Rostock, Rostock, Deutschland

**Keywords:** Hirntod, Hirntodkriterium, Postmortale Organspende, Bewusstsein, Diagnosesicherheit, Brain death, Brain death criterion, Postmortem organ donation, Consciousness, Diagnostic reliability

## Abstract

In der akademischen und öffentlichen Debatte wird der irreversible Hirnfunktionsausfall als Kriterium des Todes (Hirntodkriterium) immer wieder hinterfragt. Im vorliegenden Artikel werden 6 prototypische Thesen gegen das Hirntodkriterium diskutiert: 1) Nichtsuperiorität des Gehirns gegenüber anderen Organen, 2) Unsicherheit der Hirntoddiagnostik, 3) erhaltene Schmerzempfindung Hirntoter, 4) (spontane) sexuelle Reifung und erhaltene Reproduktionsfunktion Hirntoter, 5) Symmetrie von Hirntod und Embryonalphase, 6) Gleichsetzung des intensivmedizinisch erhaltenen Restorganismus Hirntoter mit dem lebenden Menschen.

Keine dieser Thesen hält einer kritischen Analyse stand. In Deutschland wird das Ganzhirntodkriterium angewendet. Der Hirntod geht mit dem völligen Ausfall jeglicher Empfindung, Bewusstheit, Mimik, Augen‑, Zungen- und Schlundmotorik, Willkürmotorik und Sexualfunktion einher (funktionelle „Enthauptung“). Medizinisch-technisch können andere Organe bzw. ihre Primitivsteuerung ersetzt werden, nicht aber das Gehirn. Das Gehirn, nicht der Körper, ist bestimmend für das menschliche Individuum. Die Gleichsetzung des künstlich erhaltenen Restorganismus, naturphilosophisch als lebendiges System interpretierbar, mit dem Organismus desselben lebenden Menschen wird durch die beliebige Reduzierbarkeit der Anzahl beteiligter Organe *ad absurdum* geführt. Der irreversible Hirnfunktionsausfall führt unausweichlich zum Herzstillstand, unbehandelt innerhalb von Minuten, unter Intensivtherapie i. d. R. innerhalb von Tagen. Auch beim Embryo/Fötus führt die Fehlanlage des gesamten Gehirns zum (vorgeburtlichen) Tod. Die in Deutschland gesetzliche Richtlinie zur Hirntodfeststellung hat eine im internationalen Vergleich hohe Diagnosesicherheit, es sind damit keine bestätigten Fehldiagnosen aufgetreten.

## Abstract (en)

In academic and public debate, the meaning of irreversible loss of brain function as a reliable sign of death (brain death criterion) is repeatedly challenged. In the present article, six prototypical theses against the brain death criterion are discussed: 1) the nonsuperiority of brain versus other organs, 2) the unreliability of brain death diagnostics, 3) the preserved perception of pain in brain death, 4) the (spontaneous) sexual maturation and preserved reproductive function in brain death, 5) the symmetry of brain death and embryonic stage, and 6) the equalization of an artificially respired brain-dead body and a living human being.

None of these theses withstand critical analysis. In Germany, the whole-brain death criterion is applied. Brain death involves the complete loss of all sensation, consciousness, as well as facial, ocular, lingual and pharyngeal motor, voluntary motor, and sexual function (functional “decapitation”). Other organs or their basic control can be replaced artificially, but not the brain. The brain, not the remaining body, is determinant of the human individual. The equalization of an artificially respired brain-dead organism, that may be considered as a living system from a natural philosophy point of view, and the organism of the same living human being leads, through reducibility of constituting organs, to an obvious absurdity. The irreversible loss of brain function results inevitably in cardiac arrest, spontaneously within minutes, with intensive care usually within days. In the embryo/fetus, malformation of the complete brain also results in (prenatal) death. The statutory guideline of the German Medical Association for the determination of brain death has, by comparison, high diagnostic reliability; no confirmed misdiagnoses have occurred.

## Einleitung

Die Auffassung vom Tod des Menschen als anthropologisch-kulturelles Phänomen kann je nach sozialer, religiöser, philosophischer oder bioethischer Prägung individuell und gesellschaftlich sehr verschieden sein. Dessen ungeachtet ist eine allgemeingültige Definition von Kriterien des (eingetretenen) Todes des Menschen als biologisches Wesen auf einer naturwissenschaftlich-medizinischen Basis möglich und aus verschiedenen Gründen (juristische, medizinische, alltagspraktische) notwendig. Neben den klassischen Kriterien des Todes (unaufhebbarer Herz-Kreislauf-Stillstand („Herztodkriterium“), äußere Todeszeichen (Totenflecke, Totenstarre, Verwesung etc.)) ist seit mehreren Jahrzehnten international das Kriterium des irreversiblen Hirnfunktionsausfalls („Hirntodkriterium“) anerkannt.

Zum Hirntod kommt es auf der Intensivstation, wenn infolge einer schweren Hirnschädigung ein vollständiger, unumkehrbarer Hirnfunktionsausfall eintritt, das Herz-Kreislauf-System aber durch die maschinelle Beatmung für einen gewissen Zeitraum weiter funktioniert. Der Hirntod führt unausweichlich zum Herzstillstand, ohne Beatmung innerhalb von Minuten, unter Beatmung/Intensivtherapie i. d. R. innerhalb von Tagen.

Das ärztlich-diagnostische Vorgehen zur Feststellung des irreversiblen Hirnfunktionsausfalls (Hirntoddiagnostik) ist in der seit 1982 regelmäßig aktualisierten Richtlinie der Bundesärztekammer detailliert vorgeschrieben [1]. Die Hirntodfeststellung ist in Deutschland gemäß Transplantationsgesetz (TPG) zwingende Voraussetzung für die postmortale Organspende. Daneben wird sie auf der Intensivstation auch zur Prognosebeurteilung bei akuter schwerster Hirnschädigung mit anhaltendem Koma durchgeführt. Gegenwärtig erfolgt die Hirntoddiagnostik jährlich bei ca. 2000–3000 Patienten in Deutschland, also vergleichsweise selten gemessen an der jährlichen Sterbezahl von über 900.000 in Deutschland.

Bei der Hirntoddiagnostik handelt es sich heute um die am besten dokumentierte ärztliche Todesdiagnostik, die bei richtliniengemäßer Ausführung bislang nicht zu bestätigten Fehldiagnosen geführt hat [2, 3]. Hingegen kommt es in Deutschland jährlich zu 3–10 fehlerhaften ärztlichen Todesfeststellungen anhand der klassischen Todeszeichen [4]; der Scheintod fällt dann spätestens bei der Aufbahrung oder der regulären 2. Leichenschau vor Feuerbestattung auf. Dennoch gibt es eine anhaltende Kontroverse nicht in Bezug auf die klassischen Todeskriterien, sondern nur in Bezug auf das Hirntodkriterium. Diese Kontroverse nährt sich aus verschiedenen Quellen. Zum einen gibt es teilweise Sorgen über die Zuverlässigkeit der ärztlichen Diagnostik, die durch unzutreffende, skandalisierende Medienberichte bei Einzelfällen mit bekannt gewordenen Protokollfehlern geschürt wurden (z. B. referiert in [4]).

Damit im Zusammenhang steht die gelegentlich publizierte falsche Aussage, dass bei dem künstlich beatmeten und intensivtherapierten Hirntoten eine Restempfindung, z. B. von Schmerzen, verblieben sein könnte. Tatsächlich werden nach jedem erhobenen Vorwurf der fehlerhaften Hirntodfeststellung diese Fälle von speziellen Kommissionen der Bundesärztekammer detailliert untersucht; bei allen abschließend überprüften 43 Fällen der Jahre 2010–2014 konnten die Kommissionen bestätigen, dass diese Patienten hirntot waren [4].

Zum anderen gibt es eine teils philosophisch, teils religiös, teils biologisch-ethisch geprägte akademische Debatte, deren kontroverse Positionen sich im geteilten Votum des Deutschen Ethikrates 2015 pro/kontra das Hirntodkriterium mit 18 zu 7 Stimmen widerspiegeln (Position A/Position B), auch wenn der Ethikrat einstimmig der Meinung war, dass der irreversible Hirnfunktionsausfall ausreichende Voraussetzung für die (postmortale) Organspende ist [5]. Auf Unstimmigkeiten und resultierende Dilemmata dieses Votums für das ärztliche Handeln ist hingewiesen worden [6, 7]. Dazu gehört die Unvereinbarkeit der Position B mit dem ärztlichen Tötungsverbot und der *Dead-Donor*-Regel bei der Organspende. Nachfolgend ist eine Bestätigung des Hirntodkriteriums durch die Bundesärztekammer publiziert worden [2], unter Verzicht auf eine direkte Auseinandersetzung mit den Argumenten der Position B [5], die teilweise bereits in den 1990er-Jahren Gegenstand wissenschaftlicher und medizinethischer Auseinandersetzung in Deutschland gewesen sind [5, 8–10].

Im vorliegenden Artikel werden 6 prototypische, immer wieder vorgebrachte Thesen gegen das Hirntodkriterium aus einer aktuellen neuromedizinisch-neurowissenschaftlichen Perspektive betrachtet.

## Biologie des menschlichen Gehirns

### These 1

L.S. Geisler [11]: „Hirntote sind Menschen, bei denen 97 % ihres Körpers leben, nur drei Prozent – ihr Gehirn – ist tot, ‚hirntot‘.“

### Anatomie und Energiebedarf des Gehirns

**Figure 1:**
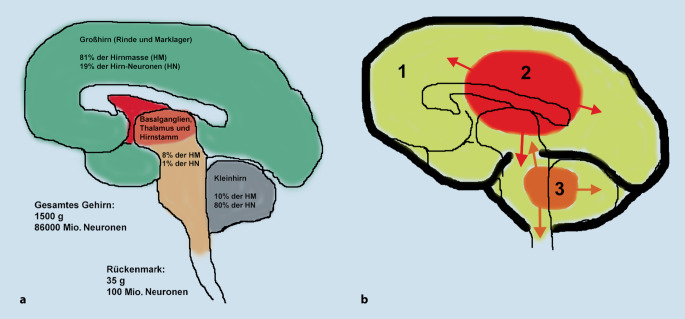

Die Zahl der Nervenzellen (Neuronen) im Gehirn ist mit 70–100 Mrd. nahezu 1000fach höher als im Rückenmark (Abb. 1a), welches größtenteils aus Nervenfasern besteht und ca. 100 (20–200) Mio. Neuronen enthält [12–14]. Im Mittel hat jede Nervenzelle im Gehirn 1000 Verbindungen (Synapsen) mit anderen Nervenzellen; jede Synapse ist veränderlich und kann wiederum selbst die Information variabel weiterleiten (synaptischer Vesikelpool); allein daraus resultiert eine enorme Dynamik der Verbindungsmatrix des Gehirns [15].

**Figure 2:**
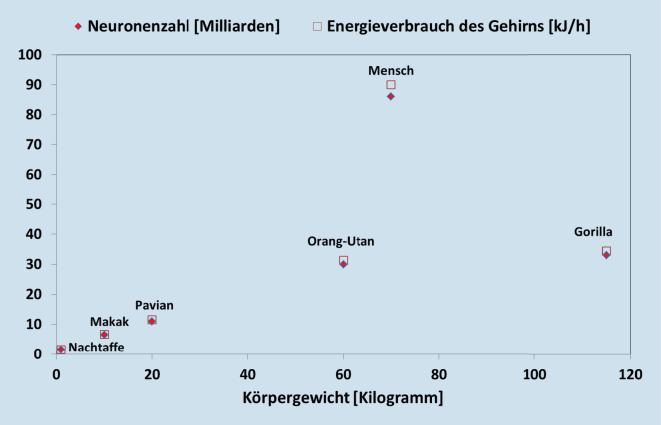

Das Gehirn des Erwachsenen macht mit durchschnittlich 1450 g zwar nur 2–3 % der Körpermasse aus, beansprucht aber 20–25 % der gesamten Energie des Körpers [12, 16]. Damit ist der Energiebedarf jedes Neurons 200‑ bis 300-mal höher als der einer durchschnittlichen Körperzelle. Bei Kindern kann der anteilige Energieverbrauch des Gehirns altersabhängig bis zu 2/3 des gesamten Energieumsatzes betragen [17]. Das Gehirn anderer Primaten wie Schimpansen und Gorillas benötigt 8–10 % der Gesamtenergie, bei anderen Säugetieren sind es unter 5 %. Der Energieumsatz des Gehirns korreliert mit der Zahl der Nervenzellen (Abb. 2) und ist dabei sehr effizient: Das menschliche Gehirn kommt mit einer Leistung von etwa 25 W aus (516 kcal/Tag; [16]). Der weltweit schnellste Supercomputer „Summit“ (Oak Ridge National Laboratory, Tennessee, USA) hat zwar eine dem Gehirn vergleichbare Rechengeschwindigkeit, aber mit 15.000.000 W einen deutlich höheren Energieverbrauch [18].

Bedeutsam ist, dass das Gehirn aufgrund seiner Sonderstellung im Organismus prioritär mit Energie versorgt wird; die Energiezufuhr wird auch unter Fastenbedingungen durch einen vom Gehirn selbst gesteuerten Mechanismus aufrechterhalten [19, 20]. Das Gehirn benötigt im Schlaf nahezu so viel Energie wie im Wachzustand, bedingt durch eine hohe intern generierte neuronale Aktivität [21]. Folglich ist die bewusste „Denkarbeit“ vergleichsweise energiesparsam, während die andauernde Grundaktivität des Gehirns und unbewusste Informationsverarbeitungsprozesse das Gros der Energie verbrauchen.

### Evolution des menschlichen Gehirns

Die erhebliche Vergrößerung des Gehirns in Relation zur Körpermasse (und zum Rückenmark) in der Evolution der Säugetiere bis zum Menschen ist mit einer gewaltigen Zunahme der Nervenzellen einhergegangen [13]. Dass das Kleinhirn adäquat mitgewachsen ist, welches vor allem der Koordination von Augen‑, Kopf‑, Rumpf- und Extremitätenmotorik dient, zeigt die enorme Bedeutung der Gesamthirngröße für die vielfältigen motorischen Kompetenzen des Menschen.

Die Gehirngröße allein ist noch nicht entscheidend. So enthält das Walgehirn trotz des veritablen Gewichts von ca. 3600 g weniger Nervenzellen als das nur 480 g wiegende Gehirn des Gorillas [12, 16]. Dies verdeutlicht die massive Zunahme der Nervenzelldichte bereits auf der Stufe der Menschenaffen. Die Kombination der Faktoren (1) Anzahl kortikaler Nervenzellen, (2) Nervenzelldichte, (3) interneuronale Distanz und (4) axonale Leitungsgeschwindigkeit bestimmt die generelle Informationsverarbeitungskapazität eines Gehirns, die in der allgemeinen Intelligenz ihren Ausdruck findet und bei dem modernen Menschen das höchste Niveau erreicht hat [22].

Der höhere Energiebedarf des Gehirns beim Menschen wurde in der Evolution kompensiert durch eine Verkürzung des Darmes, eines ebenfalls energieintensiven Organs [23]; im Gegenzug war durch die Verbesserung der planerischen und motorischen Fähigkeiten des Menschen (erfolgreichere Großtierjagd usw.) mit Einsatz des Feuers zum Nahrungsaufschluss (raschere Nahrungsaufnahme) eine energiereichere, leichter verdauliche Ernährung möglich [16, 23].

### Bedeutung des Hirnstamms

Der Hirnstamm enthält alle nervalen Leitungsbahnen, die Großhirn, Kleinhirn und Rückenmark wechselseitig verbinden (Abb. 1a). Dem Hirnstamm entspringen die Hirnnerven, welche (1) die Sinnesorgane des Kopfes (Nase, Augen, Ohren, Kopfhaut, Mund- und Rachenschleimhaut, Zunge) an das Gehirn anbinden, (2) alle Augen‑, Gesichts‑, Zungen‑, Kau‑, Schlund- und Kehlkopfmuskeln innervieren und (3) die autonome Innervation großer Teile der Eingeweide (Herz, Magen, Darm) übertragen. Im Hirnstamm sind wichtige Steuerzentren von Grundfunktionen des Organismus (Atmung, Blutdruck, Temperatur, Antrieb zur Flüssigkeits- und Nahrungsaufnahme, Sexualfunktion usw.) lokalisiert. Zudem enthält der Hirnstamm das sog. aufsteigende retikuläre aktivierende System (*Formatio reticularis*), eine Art allgemeiner Aktivator der Wachheit, dessen Ausfall zum Koma führt.

### Bedeutung des Rückenmarks

Das Rückenmark (ca. 35 g) integriert, vermittels seiner in Relation zum Gehirn kleinen Anzahl an Nervenzellen, einfache sensomotorische Reflexfunktionen (Schutzreflexe, primitive Fluchtreflexe) und übt zudem eine Relaisfunktion für die zum Gehirn aufsteigenden verschiedenartigen sensiblen Impulse aus. Die Hauptfunktion des Rückenmarks ist der Transit: (1) von motorischen Nervenimpulsen aus dem Gehirn in den gesamten Körper unterhalb des Kopfes, (2) von sensiblen Nervenimpulsen aus den Eingeweiden und dem Körper unterhalb des Kopfes zum Gehirn und (3) von Steuerungsinformationen für die autonomen Funktionen (u. a. Herz-Kreislauf-Funktion, Blasenfunktion, Sexualfunktion) aus dem Gehirn in die entsprechenden Organe. Das Rückenmark hat bei Säugetieren eine nur noch rudimentäre Eigenfunktion. Dazu gehören, neben primitiven motorischen Schutzreflexen, viszeroautonome Reflexe, denen gemeinsam ist, dass sie ganz überwiegend von verschiedenen Hirnzentren reguliert werden, etwa die Funktion der Harnblase, der Sexualfunktion und der Herz-Kreislauf-Funktion [24–26].

### Bedeutung des „Darmhirns“

Das in den letzten Jahren auch populärwissenschaftlich häufig diskutierte „Darmhirn“, also das enterische Nervensystem, ist nicht Teil des Zentralnervensystems, soll hier aber wegen der eigenständigen Steuerungsfunktionen in ein Verhältnis zum Gehirn gesetzt werden. Es enthält 200–600 Mio. Nervenzellen und liegt damit etwa in der Größenordnung der Neuronenzahl im Rückenmark. Das enterische Nervensystem spielt die Hauptrolle bei der Funktionssteuerung von Dünndarm und Dickdarm, wohingegen die Motilität des Magens und des Zwölffingerdarms vom Gehirn über den 10. Hirnnerv (Vagusnerv) kontrolliert wird. Auch die Kontrolle der Darmentleerung (Defäkation) wird dominierend vom Gehirn ausgeübt [27].

## Hirntoddiagnostik in Deutschland

### These 2

S. Müller [28]: „Daher sollte die zerebrale Angiographie für die Hirntoddiagnostik zwingend vorgeschrieben werden, in Zweifelsfällen auch fMRT, PET oder SPECT. … Die Forderung nach Verbesserung der Hirntoddiagnostik stößt selbstverständlich auf Widerstand, nicht nur weil sie steigende Kosten zur Folge hätte, sondern auch weil eine verbesserte Hirntoddiagnostik das Organaufkommen verringern könnte …“

### Entwicklung der Hirntoddiagnostik in Deutschland

Die Entwicklung der Intensivmedizin mit der Möglichkeit maschineller Beatmung führte in den 1950er- und 1960er-Jahren zu der Frage nach zuverlässigen Kriterien, die bei beatmeten Patienten mit schwerster akuter Hirnschädigung und anhaltendem Koma einen unumkehrbaren, vollständigen Hirnfunktionsausfall anzeigen. Auch in Deutschland hat es dazu, bereits vor Publikation der Harvard-Kriterien 1968 (referiert in [29]), eine intensive wissenschaftliche Auseinandersetzung gegeben [30], zunächst unabhängig von der sich ab Mitte der 1960er-Jahre in Deutschland entwickelnden Transplantationsmedizin. Die Fachdiskussion in Deutschland war stets von dem Leitgedanken getragen, dass der irreversible Funktionsausfall des gesamten Gehirns nachzuweisen ist, wobei aus anatomischen Gründen klar ist, dass mit dem Funktionsverslust von Großhirn und Hirnstamm auch der Funktionsverlust des Kleinhirns vorliegt (s. oben). Fachlich gleichsinnig wie in der BRD wurden die Kriterien zur Hirntodfeststellung in der DDR angewendet [31], auch wenn der rechtliche Rahmen der Transplantationsmedizin in den beiden deutschen Staaten bis zur Wiedervereinigung unterschiedlich war.

In anderen Ländern (z. B. Großbritannien, später teilweise USA) ist das „Hirnstammtod“-Kriterium als hinreichend akzeptiert worden [29]. Das Hirnstammtodkriterium ist prinzipiell sicher bei Vorliegen einer primären supratentoriellen (obere Schädelgrube) oder sekundären (globalen) Hirnschädigung (Abb. 1b; [29]). Jedoch kann es bei einer primären infratentoriellen (untere Schädelgrube) Hirnschädigung vor allem des Hirnstamms – wenn auch extrem selten – einen Zustand geben, bei dem weder eine Spontanatmung noch Hirnstammreflexe oder sonstige motorische Reaktionen auftreten, obwohl evtl. noch ein Bewusstsein vorliegt (*Super-Locked-in*-Syndrom; [29, 32]). Tatsächlich wurden bei solchen Patienten, die das klinische Bild des Hirnstammtodes aufwiesen, in der Elektroenzephalographie (EEG) bis zu mehrere Tage Großhirnaktivität nachgewiesen (Übersicht in [29]), aber auch noch durch visuelle Reizung evozierte Hirnrindenpotenziale [29, 33]. Diese Befunde untermauern die Notwendigkeit des Nachweises des Ausfalls der Großhirnfunktion mittels apparativer Zusatzdiagnostik bei primärer infratentorieller Hirnschädigung, was in der Aktualisierung der Richtlinie in Deutschland 1986 auf Basis des damals neuen wissenschaftlichen Kenntnisstandes konsequent umgesetzt wurde [33].

### Aktuelle Hirntoddiagnostik in Deutschland

**Figure 3:**
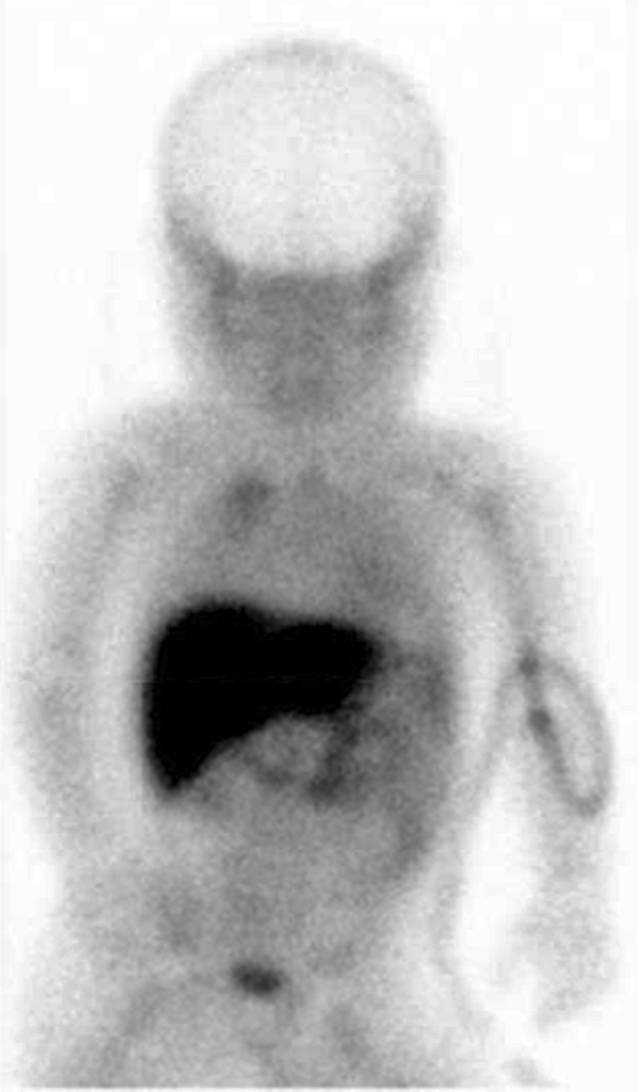

Die deutsche Richtlinie ist im internationalen Vergleich besonders streng (Infobox 1) und trägt in ihren Fortschreibungen dem jeweils aktuellen wissenschaftlichen Kenntnisstand Rechnung. Dazu erfolgt eine regelmäßige Bewertung der internationalen Fachliteratur bei den entsprechenden ständigen Gremien der Bundesärztekammer sowie der medizinischen Fachgesellschaften [1–4, 33]. Es werden nur validierte Verfahren als Zusatzverfahren in der Hirntoddiagnostik akzeptiert. Die funktionelle Magnetresonanztomographie (fMRT) und die Positronenemissionstomographie (PET) sind bislang in dieser Anwendung nicht validiert, auch wenn diese Verfahren für bestimmte Fragestellungen bei schwerer Hirnschädigung zum Einsatz kommen [34]. Hingegen sind spezielle nuklearmedizinische Verfahren (z. B. SPECT; Abb. 3) zum Nachweis des zerebralen Zirkulationsstillstandes zugelassen [1, 3]. Zur Abgrenzung eines irreversiblen Hirnfunktionsausfalls von Zuständen mit einer noch vorhandenen, auch minimalen Hirnfunktion sind die angewendeten Verfahren sicher und ausreichend [3, 29]. Die 2015 aktualisierte Richtlinie hat mit der verpflichtenden Beteiligung mindestens eines Neuromediziners mit mehrjähriger Erfahrung in der Intensivtherapie den Qualitätsstandard weiter gehoben. In ihrer Folge hat die Zahl ärztlicher Fortbildungen zu diesem Thema auf den medizinischen Fachtagungen deutlich zugenommen.

In der Praxis der Hirntoddiagnostik, die der Autor seit über 20 Jahren aus persönlicher Erfahrung überblickt, liegt heute bei allen betroffenen Patienten mindestens eine Schnittbildgebung (CT, MRT) des Gehirns vor, um den richtliniengemäßen Nachweis der schweren ausgedehnten Hirnschädigung zu führen (Ausnahmen sind möglich bei offensichtlicher Ursache, wie z. B. schwerster Schädelquetschung). Behebbare Ursachen des Hirnfunktionsausfalls werden ausgeschlossen (hirntodähnliche Erkrankungen, Medikamentenwirkungen, Stoffwechselentgleisungen usw.; [1, 35, 36]). Wenn Zweifel in Bezug auf eine medikamentöse Beeinflussung verbleiben, kommt ein Verfahren zur Feststellung des zerebralen Zirkulationsstillstandes zum Einsatz [35]. Dies gilt auch dann, wenn ein Hirnstammreflex oder der Test auf Atemstillstand nicht zuverlässig durchführbar ist [36]. Wenn an der Hirntoddiagnose auch nur geringste Zweifel bestehen, wird der Hirntod nicht festgestellt, sondern die Hirntoddiagnostik nach einem geeigneten Zeitintervall vollständig wiederholt oder ansonsten abgebrochen [3].

### Abgrenzung von hirntodähnlichen Zuständen

Ein Motiv, das in vielen Stellungnahmen von Kritikern des Hirntodkriteriums erkennbar wird, ist die Sorge, dass die Hirntoddiagnostik nicht in der Lage sei, hirntodähnliche Zustände abzugrenzen, bei denen noch ein Minimalbewusstsein bzw. eine gewisse Hirnfunktion erhalten ist [28]. Insbesondere geht es um den minimalen Bewusstseinszustand und um das Syndrom reaktionsloser Wachheit (SRW, „Wachkoma“, auch als *Persistent Vegetative State* – PVS bezeichnet). Leider kommt es in journalistischen Beiträgen immer wieder zur Nennung von Beispielfällen, die angeblich aus dem Hirntod erwacht sein sollen, bei denen aber tatsächlich kein Hirntod vorlag (und auch nicht festgestellt war), sondern ein vorübergehendes Koma nach schwerem Schädel-Hirn-Trauma (z. B. Trenton McKinley, Taylor Hale, beide USA) oder ein schweres, zunächst nicht erkanntes *Locked-in*-Syndrom (Angèle Lieby, Frankreich). Seltene Fälle einer tatsächlichen Hirntodfehldiagnose sind international berichtet worden (referiert in [3]). In Deutschland sind bei richtliniengemäßer Hirntoddiagnostik keine Fehldiagnosen aufgetreten.

## Empfindung von Schmerz

### These 3

W. Bartens [37]: „Mit feinen Messinstrumenten aufgenommene Muster des ‚hirntoten‘ Gehirns deuteten gar darauf hin, dass es auf Schmerz reagiert.“

### Empfindung und Wahrnehmung

**Figure 4:**
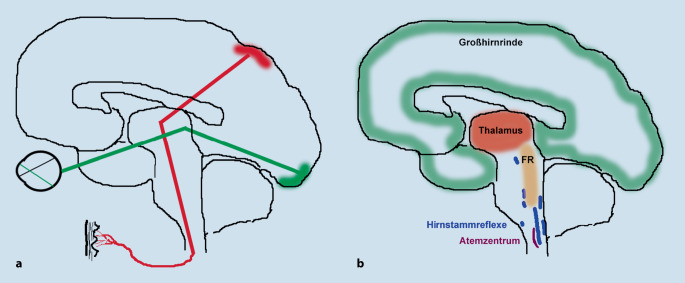

Das deutsche Wort „empfinden“ ist eine Zusammensetzung aus dem Präfix „ent-“, das meist für eine Trennung oder Entgegensetzung steht, und dem Verb „finden“ – als ursprüngliche Bedeutung gilt damit „herausfinden, wahrnehmen“ [38]. In der Physiologie wie auch in der Psychologie meint „Empfindung“ den gesamten Vorgang der direkten Aktivierung der Rezeptoren eines Sinnesorgans (z. B. Auge, Schmerzrezeptoren) als Reaktion auf einen einfachen Reiz und deren Weiterleitung an das zuständige sensorische Gehirnareal, das den Sinneseindruck produziert (Abb. 4a). „Wahrnehmung“ ist dann die weitere bewusste Einordnung der elementaren Empfindungen anhand bereits vorhandener Erfahrungen (gnostische Hirnfunktionen in den sekundären Assoziationsarealen des Großhirns). Der Physiologe R. du Bios-Reymond hat zur Erläuterung bereits 1920 geschrieben [39]: „Der Gehörsinn vermittelt deshalb Schallempfindung, weil der Hörnerv mit der Hörsphäre in Verbindung steht, der Gesichtssinn vermittelt deshalb Lichtempfindung, weil er mit der Sehsphäre des Gehirns verbunden ist.“ Neurophysiologisch sind jegliche elementare Empfindung sowie jede Bewusstwerdung von Wahrnehmungen und eigenen Gedanken an ein funktionierendes Gehirn gebunden. Dies ist durch zahlreiche elektrophysiologische Untersuchungen und funktionelle Bildgebungsstudien gut belegt (referiert in [29]). Für das Entstehen eines wie auch immer gearteten Bewusstheitsinhaltes ist das Zusammenwirken der *Formatio reticularis* des Hirnstamms, ihrer Verbindungen zum Thalamus und zur Hirnrinde und mindestens von Teilen der assoziativen Areale der Großhirnrinde notwendig (Abb. 4b; [29, 40]).

### Vegetative Reaktionen im Hirntod

Dass bei Hirntoten eine rückenmarksvermittelte vegetative Reaktion (Herzfrequenzanstieg, Blutdruckanstieg, Schwitzen) durch Reizung von Schmerzrezeptoren der Haut bzw. der Eingeweide (etwa durch chirurgische Schnitte bei einer Organentnahme) auftreten kann, ist lange bekannt [41]. Dies kann zu der Fehlinterpretation führen, dass bei dem Hirntoten noch eine Empfindung vorliege. Zur Unterdrückung rückenmarksvermittelter vegetativer und motorischer Reaktionen bei einer Organentnahme werden heute durch den Anästhesisten Opiate und Muskelrelaxanzien gegeben. Eine Gabe von Mitteln zur Schmerzausschaltung (Narkose) ist neurophysiologisch nicht sinnvoll, dies wurde durch die ärztlichen Fachgesellschaften wiederholt klargestellt. Die oben zitierte These ist unzutreffend und unbelegt [37]. Die von dem Autor, W. Bartens, angeführte Quelle, das sog. *White Paper* des President’s Council on Bioethics [42], enthält diese Aussage nicht.

## Hirntod und Reproduktionsfähigkeit

### These 4

J. Hoff, J. in der Schmitten [43]: „Hirntote Männer sind grundsätzlich erektions-, ejakulations- und zeugungsfähig, ebenso wie hirntote Frauen grundsätzlich nicht nur gebär-, sondern auch empfängnisfähig sind.“

### Hormonelle Kontrolle der Reproduktion

Das Gehirn steuert über die Hypothalamus-Hypophysen-Achse die Hormonproduktion in der Hypophyse (Hirnanhangsdrüse), der Schilddrüse, der Leber, der Nebennierenrinde, den weiblichen Ovarien und den männlichen Hoden. Eine allgemeine Bewertung von Hormonbefunden im Hirntod ist erfolgt [2]; hier soll auf die für die Reproduktion relevante Hormonregulation eingegangen werden.

Nach Eintritt des Hirntodes kommt es bei fortgeführter Beatmung und dadurch noch aufrechterhaltener Herz-Kreislauf-Funktion innerhalb weniger Tage zu einem deutlichen Abfall der Produktion des follikelstimulierenden Hormons (FSH) im Hypophysenvorderlappen [44]. Bei der Frau sind ausreichende FSH-Spiegel zwingend erforderlich für die Reifung der Eizellen in den Ovarien [45]; der FSH-Abfall im Hirntod führt somit zwangsläufig zur weiblichen (biologischen) Unfruchtbarkeit [3].

Eine besondere Situation liegt vor, wenn der Hirntod bei einer bereits schwangeren Frau eintritt. Dann muss der regelhafte Ausfall der Hypophysenfunktion mittels Hormongaben ausgeglichen werden (obligat Hydrocortison, evtl. Schilddrüsenhormone, Vasopressin), um Stoffwechsel und Herz-Kreislauf-Funktion zu stabilisieren [46, 47]. Dadurch gelang unter Intensivtherapie die ausreichende intrauterine Reifung und Entbindung des Kindes in etwa 3/4 der publizierten Fälle [47]. Soweit berichtet, ist die Entwicklung dieser Kinder in den ersten Lebensmonaten normal oder moderat verzögert, allerdings ist wenig über die längerfristige Entwicklung der so entbundenen Kinder bekannt. Die massive Ausschüttung von Östrogen durch die Plazenta bewirkt bei der (gesunden) Mutter eine Vergrößerung ihrer Hypophyse, die v. a. durch eine Vermehrung prolaktinproduzierender Zellen bedingt ist [46]. Das Prolaktin spielt eine Rolle bei der Appetit- und Blutzuckerregulation der Schwangeren und bewirkt eine Proliferation der Milchdrüsen. Da das im Hirntod in der mütterlichen Hypophyse nicht mehr gebildete Prolaktin keine Bedeutung für den Fötus oder die Plazenta hat, ist dessen Mangel nicht relevant für die intrauterine Reifung des Fötus. Zudem schüttet die Plazenta Progesteron in den mütterlichen und fötalen Kreislauf aus, das die Schwangerschaft aufrechterhält (Stopp der Menstruation, Einfluss auf das Immunsystem), aber auch wesentlich die Hirnentwicklung des Fötus beeinflusst [48]. Plazenta und Fötus übernehmen vermittels ihrer intensiven Symbiose die hormonelle Führung der Schwangerschaft [48]. Nur dadurch ist die intensivmedizinische Aufrechterhaltung der Schwangerschaft selbst nach eingetretenem Hirntod der Mutter möglich. Inzwischen ist nach gelungener Gebärmuttertransplantation (entnommen von einer hirntoten Frau) eine erfolgreiche Schwangerschaft realisiert worden [49].

Beim Mann ist während der Pubertät ein deutlicher Anstieg des FSH zur Geschlechtsreifung erforderlich. Eine (spontane) geschlechtliche Reifung hirntoter Kinder ist nicht möglich; anderslautende Berichte sind anzuzweifeln (s. unten). Beim Mann stimuliert das FSH die Proliferation der die Spermatozoen umhüllenden und ernährenden Sertoli-Zellen; FSH-Mangel führt i. d. R. zu einer Unfruchtbarkeit infolge verminderter Spermienzahl im Ejakulat (Oligozoospermie; [45]). Das in der Hypophyse gebildete luteinisierende Hormon (LH) hat eine noch entscheidendere Rolle bei der Spermatogenese, und zwar über die Stimulation der Testosteronproduktion in den Leydig-Zellen im Hoden. Im Hoden liegen 50- bis 100fach höhere Testosteronkonzentrationen als im Blut vor; ohne eine ausreichende Testosteronkonzentration erliegt die Spermatogenese vollständig [45]. Nach Eintreten des Hirntodes fallen die Spiegel von LH und Testosteron zwar ab, können aber noch im Normbereich bleiben, zumindest für einige Tage [44, 50]. Dies lässt theoretisch die Möglichkeit offen, dass bei Hirntoten für eine gewisse Zeit eine schwache Spermatogenese fortbestehen könnte; nach Kenntnis des Autors liegen dazu keine wissenschaftlichen Untersuchungen vor. Nach dem Funktionsverlust des Hypothalamus wird die hypophysäre Restproduktion von LH durch Gonadoliberin stimuliert, das wahrscheinlich aus den Baucheingeweiden stammt [44, 51].

### Neuronale Kontrolle der Sexualfunktion

Die Sexualfunktion der Frau und des Mannes unterliegt einer komplexen Steuerung durch das vegetative (sympathische, parasympathische) und somatische (sensible, motorische) Nervensystem. Die penile Erektion und Ejakulation des Mannes erfordern ein perfektes Zusammenspiel von Strukturen im Gehirn, Rückenmark und peripheren Nervensystem [26, 52]. Im Hirntod sind eine eigenständige penile Erektion und Ejakulation nicht möglich. Es kann aber innerhalb der ersten Tage nach Eintritt des Hirntodes durch eine Elektrostimulation mittels anal in den Mastdarm eingeführter Elektroden eine partielle Ejakulation zur Samengewinnung auslösbar sein [53]. Alternativ ist eine chirurgische Samenextraktion aus dem Hoden bei Hirntoten wie auch bei klassisch am Herzstillstand Verstorbenen durchgeführt worden [53, 54]. Ein selten beobachteter Priapismus (pathologische penile Dauererektion) bei Hirntoten wie auch bei klassisch (i. d. R. durch Erhängen) Verstorbenen wird auf eine Kompression des unteren Hirnstammes und oberen Rückenmarks zurückgeführt [55].

## Vergleich von Hirntod und Embryo

### These 5

D. Birnbacher [56]: „Ein weiterer anthropologischer Einwand gegen eine Gleichsetzung von Hirntod und Tod ist die sich aus der Gleichsetzung ergebende Asymmetrie …: Der Hirntote gälte als tot, der Embryo oder Fötus dagegen als lebendig, beide befinden sich jedoch strukturell in derselben Lage.“

### Ontogenese von Herz und Gehirn

Der philosophische Vergleich des Hirntoten (mittels Beatmung erhaltene Herz-Kreislauf-Funktion) mit dem Embryo bzw. Fötus ist nur dann angemessen, wenn sich dieser auf das Stadium bezieht, ab dem das Herz des Embryos anfängt zu schlagen; tatsächlich wird dies auch explizit so diskutiert [57]. Im Laufe der Entwicklung ist das Herz-Kreislauf-System das erste funktionsfähige System des Embryos. Der Herzschlag des menschlichen Embryos beginnt am 22. Tag nach der Befruchtung (Embryonalstadium 10 nach Carnegie; 4–12 Somitenpaare; Länge des Embryos: 2 mm; Fotografie; siehe [58]). Zu diesem Zeitpunkt ist bereits die sog. Neuralrinne angelegt und der rostrale Anteil, der sich zum Gehirn auswächst, klar abgegrenzt [59]. Störungen des Neuralrohrschlusses zu diesem Zeitpunkt führen zu schwerster Kopf- und Hirnmissbildung (*Kraniorhachischisis totalis*), die mit dem Leben unvereinbar ist und immer zur Totgeburt führt [60]. Ebenfalls in Symmetrie zum Hirntodkriterium kann eine Schädigung bzw. Entfernung des Gehirnanteils der Neuralleiste ab dem Embryonalstadium 10 nicht mehr repariert werden und führt zum vorgeburtlichen Tod [59, 61].

An dieser Stelle soll betont werden, dass die tödliche *Kraniorhachischisis totalis* abzugrenzen ist von der sog. Anenzephalie (unvollständige Gehirnanlage), die aus einer Störung der Hirnentwicklung in einem späteren Embryonalstadium resultiert [59]. Diese Kinder haben zwar eine begrenzte Lebenserwartung (i. d. R. Versterben innerhalb der ersten Lebensmonate), soweit sie überhaupt lebend geboren werden. Sie können aber vereinzelt durchaus zu mimischen Reaktionen (z. B. Lächeln) und Stimmentäußerungen (Gurren) in der Lage sein [62]. Es besteht in Deutschland ärztlicher und gesellschaftlicher Konsens, dass anenzephale Kinder – wie jeder andere Mensch – nicht für eine Organentnahme wenige Minuten nach Herz-Kreislauf-Stillstand in Betracht zu ziehen sind, was in anderen Ländern jedoch anders gesehen wird (*Non-heart-beating-donor*-Konzept; [63]).

## Organismus und Individuum

### These 6

R. Stoecker [64]: „Zu deutlich ist die Diskrepanz zwischen der Hirntod-Konzeption und dem äußeren Anschein von Lebendigkeit hirntoter Patienten: Atembewegungen, die rosige Färbung der Haut, Körperwärme, Bartwuchs, Wundheilung, regelmäßige Körperausscheidungen und gelegentliche Bewegungen der Extremitäten, aber auch unter Umständen Schwangerschaft, Körperwachstum und sexuelle Reifung, das sind traditionell eigentlich alles Lebenszeichen, …“

### Organismische Funktionen bei Hirntod

Im hirntoten Körper, dessen Herz-Kreislauf-Funktion mittels Beatmung für einen begrenzten Zeitraum aufrechterhalten werden kann, bestehen die folgenden Funktionen mehr oder weniger lange fort: Aufrechterhaltung der Körpertemperatur, Erholung von Infekten (ggf. unter antibiotischer Therapie), spinale Reflexe, Haar- und Nagelwachstum, Verdauung und Nahrungsresorption im Darm, Detoxikation des Blutes in Leber und Nieren, Urinproduktion, Stuhlentleerung, Wundheilung, Aufrechterhaltung der Schwangerschaft. Die genannten systemischen Funktionen sind gemäß Position B des Deutschen Ethikrates [5], die wesentlich durch D.A. Shewmon und das *White Paper* inspiriert ist [42, 65], als Ausdruck eines „intakten Organismus“ und damit eines „noch lebenden“ Menschen anzusehen. Dabei wird auf die Vielzahl („litany“ [65]) systemischer Funktionen abgehoben, um den Hörer/Leser zu überzeugen. Allerdings sind einige „Lebenszeichen“ unzutreffend bzw. für einen Laien irreführend formuliert [5, 64].

Unzutreffend ist zunächst die ursprünglich von D.A. Shewmon stammende pauschale Aussage, dass hirntote Kinder sexuell reifen würden. In keinem der beiden Fälle („Baby A“, „BES“), auf die sich D.A. Shewmon beruft [65, 66], ist eine korrekte Hirntoddiagnostik erfolgt. So traten bei „Baby A“ im vermeintlichen Hirntod (mit berichteter vorzeitiger Entwicklung von Schamhaaren und einer Ejakulation im Alter von 1 Jahr) noch spontane Atembewegungen während des Apnoetests sowie Fluchtreaktionen der Extremitäten bei Schmerzreizen auf, die die beteiligten Ärzte als spinale Reflexbewegungen fehlinterpretierten [66]. Bei dem 13-jährigen Jungen „BES“ (mit berichtetem leichten Wachstum von Penis und Schamhaaren im Verlauf von 2 Monaten) erfolgte der Apnoetest mit völlig unzureichendem Anstieg des p_a_CO_2_ nur bis in den Normalbereich; zudem zeigte das Kind Kopfwendebewegungen [66], die aufgrund ihrer unsicheren Zuordnung als spinaler Reflex zwingend den Nachweis des zerebralen Zirkulationsstillstands erfordert hätten [36]. Dies sind die beiden einzigen Fälle mit berichteten Zeichen sexueller Reifung aus seiner Fallsammlung „Langzeit-Hirntoter“ [65, 66]. Ein anderer Fall („TK“, ohne sexuelle Reifung) ist in einer späteren Publikation näher beschrieben worden [67]; erst darin wird klar, dass bei diesem 4½-jährigen Kind keine vollständige Hirntoddiagnostik erfolgte (fehlender Apnoetest) und später – entgegen dem Erstbericht [66] – sogar eindeutige neurophysiologische Zeichen einer Hirnstammaktivität nachweisbar waren (erhaltene P14-Welle in den somatosensibel evozierten Potenzialen; [1, 67]). Auch viele andere Langzeithirntote, auf die D.A. Shewmon verweist [65, 66], sind nicht als gesichert Hirntote anzusehen, nicht einmal nach US-amerikanischen Richtlinien. Zudem ist bedeutsam, dass selbst im Falle einer erfolgten Hirntoddiagnostik die US-amerikanische Richtlinie bei Kindern erheblich von der strengeren deutschen Richtlinie abweicht [3]. Insofern ist zu kritisieren, dass prominente Akademiker die These, eine (spontane) sexuelle Reifung sei im Hirntod möglich, in ihre öffentlichen Positionen zum Hirntodkriterium in Deutschland übernommen haben [5, 64].

Potenziell irreführend ist die isolierte Formulierung „unter Umständen Schwangerschaft“ [64] bzw. „erfolgreiche Schwangerschaften“ [5], da diese ohne weitere Erläuterung impliziert, dass eine hirntote Frau schwanger *werden* könnte, was nur bei verwerflicher und massiver medizinischer Manipulation denkbar ist (s. oben). Eine bei Eintritt des Hirntodes bereits bestehende Schwangerschaft kann unter intensivmedizinischer Aufrechterhaltung des Herz-Kreislauf-Systems mittels Beatmung teilweise zu einer erfolgreichen Entbindung geführt werden, wobei aber die Entwicklung eines Kindes im Mutterleib von der Plazenta gesteuert wird (s. oben). Irreführend ist die Formulierung „Atembewegungen“ [64], da der Hirntote keine Eigenatmung mehr hat, sondern maschinell beatmet wird. Irreführend ist die Formulierung „gelegentliche Bewegungen der Extremitäten“ [64], denn diese impliziert, dass noch willkürliche Bewegungen auftreten könnten. Tatsächlich können im Hirntod gelegentlich rückenmarksgenerierte, monotone, langsame Reflexbewegungen auftreten, die zeitlich befristet sind und anhand ihrer Muster klar von den Bewegungen Nichthirntoter abgrenzbar sind [36].

Nach K. Steigleder zwingt uns die Beobachtung organismischer Funktion beim Hirntoten dazu, deutlicher zu fassen (und nicht neu zu definieren), worin der Tod des Menschen eigentlich besteht: Voraussetzung leibhafter Existenz ist Empfindungsfähigkeit; insofern Empfindungsfähigkeit an ein funktionsfähiges Gehirn gebunden ist, bedeutet der vollständige irreversible Hirnfunktionsausfall das irreversible Ende des leibhaft existierenden Organismus, also seinen Tod [68].

### Welche Körperteile machen ein Individuum?

Zunächst ist die Frage zu betrachten, welche Teile mindestens das menschliche Individuum ausmachen. Die minimierende Anwendung der Sichtweise der Position B (Deutscher Ethikrat [5]) führt zum Paradox: Es ist nicht zu beantworten, wie viele Organe bzw. Körperteile nach Eintritt des irreversiblen Hirnfunktionsausfalls theoretisch entfernt (oder künstlich ersetzt) werden können, bevor ein unter Intensivtherapie funktionierendes Organsystem nicht mehr als lebender Mensch bezeichnet werden kann.

Dann ist die Frage zu behandeln, ob das Gehirn „bestimmend“ oder „nicht bestimmend“ ist für die Zuweisung der Eigenschaft, ein lebender Mensch zu sein. Der Hirntod geht mit einer funktionellen „Enthauptung“ einher (F. Erbguth: „intrinsische Enthauptung“ [4]). In Anlehnung an P. Lee können dann folgende Fälle betrachtet werden [69]. Einzelnen Körperteilen (z. B. Gliedmaßen, Einzelorganen) einer Person mit dem Namen „X“ wird man nicht das Attribut, ein menschliches Individuum zu sein, zuweisen. In dem theoretischen Extremfall, dass es möglich wäre, den Kopf vom übrigen Leib zu trennen und beide Teile getrennt am Leben zu erhalten (z. B. mittels künstlichen Ersatzes der fehlenden Hälfte), würde man das Attribut menschliches Individuum („X“) nur dem natürlichen Kopf (mit künstlichem Körper), aber nicht dem natürlichen Körper (mit künstlichem Kopf) zuweisen. Für Position A (Deutscher Ethikrat) wird zu Recht konstatiert: Das Gehirn ist das einzige Organ, mit dem die vorherige personale Identität unterginge, wenn es technisch substituiert oder ersetzt würde [5].

**Figure 5:**
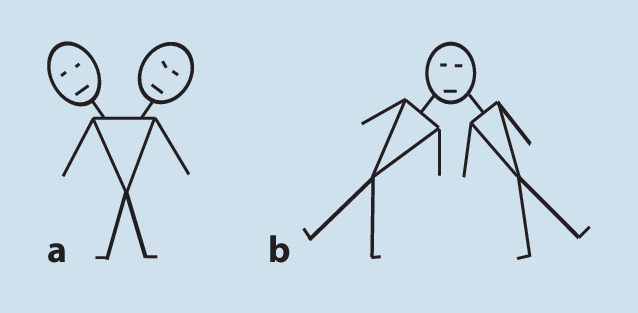

Die Frage, ob der Kopf oder der (übrige) Körper das Individuum bestimmt, kann auch mit einem Blick auf die Ansprache siamesischer Zwillinge durch ihre Mitmenschen beantwortet werden. Bei siamesischen Zwillingen ist es in der vorgeburtlichen Entwicklung zu einem teilweisen Zusammenwachsen ihrer Körper gekommen. Siamesische Zwillinge mit extremen Ausprägungen, die für die obige Frage bedeutsam sind (Abb. 5), sind sehr selten und daher v. a. in historischen, oft bewegenden Darstellungen und Berichten über ihre Schicksale zu finden [70, 71]. Diese zeigen, dass schon im traditionellen Verständnis der Kopf entscheidend das Individuum bestimmt.

### Allgemeine Definition von Leben

Neben den vielfältigen Anschauungen zum Phänomen „Tod“ gibt es zahlreiche Definitionen von „Leben“ und zu diesen eine vielstimmige Diskussion [72]. Die folgenden essenziellen Eigenschaften individueller Lebewesen sind aber breit akzeptiert: funktionelle Autonomie und Selbstreproduktion [72]. Bei getrennter Betrachtung beider Lebensmerkmale kann „funktionelle Autonomie“ eventuell noch für den beatmeten Körper eines Hirntoten gelten, da dieser zu einigen systemischen Funktionen in der Lage ist [5, 65]. „Selbstreproduktion“ ist beim intensivtherapierten Hirntoten nur auf zellulärer Ebene gegeben (Zellteilung), nicht auf organismischer Ebene (s. oben). Erst recht in ihrer Kombination sind diese beiden Lebensmerkmale nur möglich für einen Menschen (hier gemeint in der Verallgemeinerung), der sein Reproduktionsverhalten selbst bestimmt, sowohl im negativen (Entscheidung gegen die Zeugung eigener Kinder) wie im positiven Sinne (selbstbestimmte Partnerwahl bei der Zeugung von Nachkommen; [73]). Diese Selbstbestimmung ist ohne Hirnfunktion nicht möglich. Insofern sprechen auch die o. a. essenziellen Merkmale von Leben bei integrativer Betrachtung gegen die Annahme, ein hirntoter Mensch sei ein lebender Mensch.

## Fazit

Die hier diskutierten Thesen gegen das Hirntodkriterium sind mittels naturwissenschaftlich-medizinisch nachprüfbarer Befunde zu entkräften. Das Hirntodkriterium ist valide.

### Infobox 1 Vorgaben der Bundesärztekammer zur Feststellung des irreversiblen Hirnfunktionsausfalls, die die besonderen Qualitätsstandards in Deutschland widerspiegeln (modifiziert nach [3])

- Verbindlicher Facharztstandard bei allen an der Untersuchung beteiligten Ärzten
- Strikte Unabhängigkeit zwischen hirntodfeststellenden und transplantierenden Ärzten
- Feststellung des irreversiblen Hirnfunktionsausfalls durch mindestens 2 Ärzte
- Spezielle Vorgaben zur Zusatzqualifikation der jeweils beteiligten Ärzte in der Intensivmedizin, Neuromedizin, Pädiatrie bzw. Radiologie
- Nach Feststellung der klinischen Symptome des Ausfalls der Hirnfunktion immer Nachweis der Irreversibilität mit einer weiteren Untersuchung (je nach Alter des Patienten und Art der Hirnschädigung: apparative Zusatzdiagnostik und/oder zweite klinische Untersuchung nach definierter Beobachtungszeit)
- Bei primärer infratentorieller Hirnschädigung obligate apparative Zusatzdiagnostik
- Nichtanwendbarkeit des Apnoetests bei chronischer Adaptation an erhöhte p_a_CO_2_-Werte (z. B. bei chronisch obstruktiver Lungenerkrankung Stadium 3 oder 4 nach Gold); in diesen Fällen obligater Nachweis des zerebralen Zirkulationsstillstands
- Bei der Elektroenzephalographie wiederholtes Setzen von (nichtverletzenden) Schmerzreizen
- Ausschließliche Verwendung (validierter) lipophiler Radiopharmaka bei der Perfusionsszintigraphie mit besonders hohen Qualitätsanforderungen zum Nachweis des zerebralen Zirkulationsstillstands

## Interessenkonflikt

U. Walter ist Mitglied des Ständigen Arbeitskreises „Richtlinie zur Feststellung des irreversiblen Hirnfunktionsausfalls“ des Wissenschaftlichen Beirats der Bundesärztekammer, Vorsitzender der Hirntodkommission der Deutschen Gesellschaft für Klinische Neurophysiologie und Funktionelle Bildgebung (DGKN) und Mitglied der Kommission „Irreversibler Hirnfunktionsausfall“ der Deutschen Gesellschaft für Neurologie (DGN). Der Autor hat Honorare für konsiliarärztliche Tätigkeit von der Deutschen Stiftung Organtransplantation (DSO) erhalten.
